# Tuberculosis control from the perspective of health professionals working in street clinics

**DOI:** 10.1590/1518-8345.2691.3095

**Published:** 2018-11-29

**Authors:** Paula Hino, Aline Aparecida Monroe, Renata Ferreira Takahashi, Káren Mendes Jorge de Souza, Tania Maria Ribeiro Monteiro de Figueiredo, Maria Rita Bertolozzi

**Affiliations:** 1 Universidade Federal de São Paulo, Escola Paulista de Enfermagem, São Paulo, SP, Brazil.; 2 Universidade de São Paulo, Escola de Enfermagem de Ribeirão Preto, PAHO/WHO Collaborating Centre for Nursing Research Development, Ribeirão Preto, SP, Brazil.; 3 Universidade de São Paulo, Escola de Enfermagem, São Paulo, SP, Brasil.; 4 Universidade Estadual de Campina Grande, Departamento de Enfermagem, Campina Grande, PB, Brasil.

**Keywords:** Community Health Nursing, Public Health, Tuberculosis, Homeless Persons, Vulnerable Populations, Health Vulnerability

## Abstract

**Purpose:**

to present the opinion of professionals about street dwellers undergoing treatment of tuberculosis and identify strategies of control of tuberculosis in this population.

**Method:**

an exploratory and descriptive study involving 17 health professionals working in street clinics. A semi-structured study composed of closed questions and a guiding question. The statements were analyzed using the discourse analysis technique, resulting in the identification of two analytical categories: 1. Meanings attributed to street dwellers with tuberculosis, and 2. Control of tuberculosis in homeless people.

**Results:**

the analysis identified situations that limited adherence to tuberculosis treatment, including the reasons for staying in the streets, living conditions, and risk factors (dependence on alcohol and other drugs, short-sightedness, constant relocations, and lack of perspectives). Street dwellers were knowledgeable about the disease. Furthermore, there were difficulties in solving several problems of people living in the streets, including living conditions and lifestyle, social stigma, relocations, drug abuse, and lack of life project.

**Conclusion:**

coping with the complexity of situations related to living in the streets limits to the work of health professionals because these situations go beyond health care and require intersectoral actions.

## Introduction

Street people belong to a social group that makes temporary or permanent use of public places as living spaces. These people are socially excluded, cannot satisfy basic needs, and live in the line of indigence or absolute poverty^1^.

In 2015, 15,905 people were in a street situation in the city of São Paulo, of which 82% were men, 51.9% were aged 18 to 49 years, and 24.4% were older than 50 years. Approximately 50% of this population (7,335) lived exclusively on the streets, primarily downtown, and the remaining population lived on the streets but slept in shelters^2^.

The living conditions and social exclusion of this group increase its vulnerability, especially regarding the health-disease process. For this reason, in 2011, the Ministry of Health defined guidelines for the organization, operation, and training of street clinic teams to thoroughly meet the unique needs of this population^3^.

Street people are more vulnerable to tuberculosis (TB) because of social and health conditions, and the risk of illness in this population is 48 to 67 times higher than that in the general population. Nutritional deficiencies, use of alcohol and other drugs, sleep deprivation, lack of safety, HIV infection, old age, and limited access to health care impair immune function and increase the likelihood of developing TB^4^ ^-^ ^5^.

The rate of TB in this group, corresponding to 2,445.8 cases per 100,000 inhabitants in the city of São Paulo in 2015, is approximately 48 times higher than that in the general population (51.1 cases per 100,000 inhabitants)^1^. As a neglected disease with limited social visibility, TB represents an aggravation of the health-disease process. Therefore, the control of TB in this social group is challenging because of their living conditions and lifestyle. Studies on TB in homeless people evaluated adherence to treatment^6^, outbreaks and risks^7^ ^-^ ^11^, and care actions^5^.

In view of the specificities and challenges for controlling TB in street dwellers, the objective of this study is to present the opinions of health professionals about homeless people undergoing TB treatment and identify strategies for disease control in this group.

## Methods

This exploratory, descriptive, qualitative study involved 17 health professionals, including two physicians, three nurses, eight community health agents, two nursing assistants, one social worker, and one social agent from the street clinic team of a Primary Health Care Unit (PHCU) in the central region of São Paulo. The inclusion criterion was working for at least 3 months in a street clinic because of the increased experience in caring for homeless people. Of the 25 professionals who composed the team, four refused to participate in the study, three did not meet the inclusion criteria, and one was on medical leave, totaling a sample of 17 subjects. The data were collected from November to December of 2016 via individual 25- to 50-min interviews held in a reserved room of the PHCU to guarantee data privacy and confidentiality. The interviews were conducted by the first author, who had no relationship with the interviewees. The interviews were guided by a semi-structured questionnaire with questions on sociodemographic characteristics and the following guiding question: “Report the experience of caring for street dwellers with TB.”

The testimonies, recorded digitally, were transcribed and analyzed using the speech analysis technique by extracting statements that supported the construction of two analytical categories: 1. “Meanings attributed to homeless people with TB” and 2. “Control of TB in homeless people.” This technique allows decoding speeches that reflect the opinion of interviewed subjects by identifying themes and figures^12^. The interviews were analyzed using the Theory of Social Determination of the Health-Disease Process.

The speeches were analyzed in each professional category and were identified with Arabic numbers according to the sequence of interviews.

The study complied with the ethical principles of Resolution 466/2012 and was approved by the Research Ethics Committee of our institution under Opinion No. 1.553.500.

## Results

Most interviewed professionals were women (n = 14), aged 30-39 years (n = 7), 50-59 years (n = 7), or >60 years (n = 3). Eight professionals were single, six were married, and three were divorced. The educational level included complete higher education (n = 6), complete secondary education (n = 10), and complete primary education (n = 1).

The analysis of the two analytical categories allowed understanding the opinion of health professionals working in street clinics about street dwellers and the strategies for managing TB in this social group. The first analysis focused on the reasons for living in the streets, living conditions, and risk factors.

With respect to the category “Meanings attributed to street dwellers with TB, ”the health professionals considered that the life history of these people indicated that living on the street was not a choice but the result of a set of circumstances that led to family breakup: *Everyone has a story, no one went to the street because he wants or likes to stay on the street, it is because of family breakup, divorce, or disappointment* (health worker No. 2).

The reasons most cited by the professionals were family breakup and immigration. The causes of the family breakup were abuse of alcohol and other drugs, mental problems, inability to meet the basic needs of the family because of lack or insufficiency of financial resources, and having a sexual orientation not accepted by the family.

It was evident that some street people did not want to return to family life, and individuals who wanted to return to the family had difficulties reestablishing the trust of the family. There were cases of family reconciliation in which, although the person had returned to the family, the disappointment of the parties involved did not allow them to continue together, as these testimonies indicate: *I have been living in the streets for 40 years, and you do not say that I am in the street, I got used to the life of a homeless* (health worker No. 8). *... there are people who have families but want to live another kind of story, feel free on the street, and they say: “Here I do not have to pay bills, live by rules, I go where I want.” And there are people who fought to get off the streets, could not do that, and remained in the streets* (nurse No. 1).

Living conditions are precarious for people living in public places or invaded buildings: *It is better to live on the street because these invaded buildings are horrible: they lack basic sanitation and sewers are open* (nurse No. 2).

The constant relocations of populations that become indigent may be related to conflicts between groups and the fact that these places are characterized by the type of illegal drug used by dwellers. The dwellers who do not want to undergo treatment avoid remaining in a single place for a long time to avoid being found by health professionals. The same happens when there is the fear of being identified by the police because many people living in the streets are fugitives from the police.

Other characteristics of this population are short-sightedness and difficulty in complying with rules because this population wants immediate solutions to their needs. The work dynamics of street clinics, in which professionals seek and provide individual health care, may contribute to this characteristic: *...because they think we are after them, we insist that they take care of themselves, then they think: “They will have to find a way for me to get me in front of everybody.” Most do not wait and walk away* (health worker No. 8).

Another difficulty reported by professionals was the delay in having access to hospital care because of the disarticulation of the healthcare network, as illustrated in the following testimony: *By the time you find a vacancy, the patient is no longer willing to be hospitalized. The difficulty is offering available services daily but they are not available when you need it, and you have to wait. When the vacancy is available, these people no longer want it* (health worker No. 1).

The second category-TB control in homeless people-analyzed the knowledge of homeless people about TB, and the conditions and limitations of TB control actions. Street people have adequate and sufficient knowledge about TB either because they have had the disease or because they know someone who was treated or died because of it: *Today, on the streets, when you try to explain what TB is, they give you a lecture* (health worker No. 1).

However, people living in the streets have shown no interest in health care and have been indifferent to life or the possibility of having TB. The diagnosis of TB did not change the behavior of this population, who responded with negation or absence of reaction: *For them, it does not matter, it is as if life no longer makes sense, then having a disease does not make any difference. They often say, “Dying is a bonus”* (health worker No. 8).

The control of TB in homeless people begins with the active search for symptomatic individuals in the territory, including those with signs and symptoms, history of diagnosis of TB and/or previous treatment, and collection of pulmonary secretions for analysis: *Sometimes, you say “Good morning” and the person responds by coughing, putting his hand in the mouth. We take the opportunity to ask how long he has been coughing, if he had a fever or excessive sweat. In the approach, it is part of the ACS protocol to investigate TB. The orientation is that coughing for at least 3 weeks should be investigated, in our case, because of the street condition, 2 weeks is enough to request sputum collection* (health worker No. 1).

Directly Observed Treatment (DOT), depending on the conditions of the patient, can be performed in the PHCU or in the street clinic at any time, as one professional reported: *I was tired of searching during work hours and when I was on the subway to go home, I found some homeless people. So I took medicine in the bag and treated them when I was coming home* (nurse No. 1).

Establishing a bond with street dwellers with TB is essential for treatment. In general, the indigents were resistant to interaction on the first contact; however, over time, they accepted the approach of the professionals and began to interact: *It is not from one day to the other, we gain space gradually. In the first day, you go by and say ‘Good morning,’ and the person turns away, but on another day, you do the same thing and gradually gain their trust* (health worker No. 7).

Professionals reported that street people recognized the work of street clinics teams as beneficial for bonding and trust: *The person in the blue jacket is recognized, they say: “The angels have come!” They value us a lot and know that if there is someone ill and a health professional is not available on the street, you have to go to the primary health care unit* (social agent No. 2).

The factors that compromise adherence to treatment in this population include constant relocations, abuse of alcohol and other drugs, lack of perspective for the future, and lack of a life project: *... because they do not stay in one place, in the morning they are here, in the afternoon they go there, tomorrow they change. It is difficult for them to be in the same place, and if you tell them to come and take the medication, they come one day but do not come the other, and so do not finish treatment* (health worker No. 2).

The abuse of psychoactive substances is common in the street population and compromises individual health, effectiveness of drug treatment, and elaboration of a life project: *... they seem to have lost their will to live, do not dream anymore, it is much easier to make a follow-up in the morning than in the afternoon because they are drunker in the afternoon* (social agent No. 3).

The irregular use of medications extends treatment for a period longer than 6 months, favoring treatment discontinuation: *We were going to give the medication in the morning, but he was asleep because of the consumption of illicit drugs, and in this case, we do not wake up. That’s why he was treated for one additional month. We would come back in the afternoon, and he would be either sleeping or away because he works as a recycler in the afternoon* (health worker No. 7).

Other difficulties in managing TB were failure to accept the disease and therapy, prolonged treatment time, side effects of medications, lack of a treatment schedule, and poor living conditions: *At first, it is difficult to comply with treatment, treatment is performed correctly for one week but in the following week, treatment is skipped for 2 to 3 days, and then we talk about the importance of treatment* (social agent No. 1). *Sometimes you get there, and they have not eaten or drank anything and so cannot take the tablet* (health worker No. 2).

The constant relocations of homeless people hinder the continuity of treatment; however, some channels of communication among street clinic teams allow locating these people: *We have an e-mail and a WhatsApp group for exchanging information: this person disappeared, interrupted treatment or was found in a specific region* (nurse No. 3).

The reception centers offer access to shelters and better conditions for undergoing treatment; however, many homeless people prefer remaining on the street full time, claiming that there is a higher risk of being stolen and assaulted in shelters because of the abuse of alcohol and other drugs, although their use is banned.

Discrimination was evidenced by the discouragement of health professionals for working with this social group: *We are often victims too, our colleagues say: “Your job is nothing, it is investment lost, it is drying ice, what you do will lead nowhere.” So if we, as health professionals, suffer prejudice from other colleagues, imagine the user* (physician No. 1).

The health professionals reported other difficulties, including inadequate work conditions and care-related risks: *I need to have more resources to go to the street, I do a bandage on the street, but what about environmental contamination? We have to clean the wound, and where do this water and garbage go to?* (nurse No. 3). *There is no help for sputum collection, and we are working to identify a bacterium but may be at risk of being contaminated. In addition, if I am in a drug trafficking area and a conflict occurs among homeless people, how should I react as a professional, call the police? If I work with the police, I cannot approach homeless people* (health worker No. 1).

If performed in an inconsistent manner, the DOT requires hospitalization in a rehabilitation hospital of another municipality as a complementary approach. In this case, the street clinic team monitors treatment until hospital discharge and patient return to the territory. One of the interviewed professionals considered that people living in the streets should be retreated compulsorily because of the possibility of transmission of the Bacillus: *If the patient abandoned TB treatment once, he has to be detained to allow adequate treatment, and you cannot say, “Stop breathing because you have TB”* (nurse No. 2).

Indigents who perform DOT regularly receive a monthly basic food staple as an incentive for treatment adherence. However, the use of this benefit is not always adequate because of the difficulty of this population in preparing food, and the food staple is exchanged for other needs or desires. An alternative approach was to accredit restaurants to provide meals.

Health services and social equipment such as reception centers are insufficient to meet the demands of homeless people because of the increase in this population. In this respect, the type of care provided by street clinic teams facilitates the access to health care resources because this population can receive appropriate care in their territory, and a bond can be established with health professionals.

There are several conveniences for TB control among street dwellers related to the organization of health services, including the possibility of having consultations with physicians and nurses either with or without an appointment. Therefore, the health professional is available to provide care when the individual seeks the service. Another convenience is a vehicle to transport people with mobility limitations between health services such as a consultation with a medical specialist or an examination in a place distant from the territory in which the patient lives. Other facilitating conditions were the collection of sputum in the territory where the patient lives and the possibility of hospitalization in a rehabilitation hospital for individuals with difficulty in adhering to the individual treatment plan.

The increased awareness and instrumentalization of PHCU workers to care for homeless people were considered critical attributes, which qualified these professionals for the humanized care of this population: *Today we have an excellent team, the professionals who worked in the beginning left, so the responsiveness has improved. The importance of humanization was discussed extensively at meetings, and those who did not adapt eventually left* (social agent No. 3).

The treatment of TB in indigents exceeds the dimension of the disease, and therefore it is essential to inform them about the importance of treatment and damage mitigation, facilitating access to health care services and social equipment. Considering the chain of disease transmission, epidemiological surveillance is indispensable and demands the constant search for individuals and close contacts with diagnosis and/or under treatment in the territory because of the possibility of transmission of the bacillus.

The provision of vocational courses and/or job opportunities for people living in the streets can change their social status: *... they could have manual work to take them out of the streets, contact the families or find a place for the homeless to live because most of them return to the street when treatment ends, which limits the effectiveness of our actions* (health agent No. 5).

## Discussion

The speeches of the interviewed professionals revealed some characteristics of street dwellers, including the abuse of alcohol and other drugs, constant relocations, short-sightedness, and lack of self-esteem, and these characteristics compromise health care and adherence to treatment of TB. Similar reasons were found in studies conducted in other regions^5^ ^,^ ^13^ ^-^ ^14^.

These results corroborate those of the literature on the reasons why people live on the streets, including the search for freedom, family breakup, dependence on alcohol and illicit drugs, mental illness, constant relocations, unemployment, poverty, and personal disappointment^13^ ^-^ ^15^. The reasons are diverse and involved social exclusion (deliberate or not).

Living on the street sets a new phase in a person’s life, where privacy no longer exists. The analysis of the narratives indicates that the experience of living on the street is complex because some individuals reported being willing to return to the family whereas others became accustomed to living in the streets and did not wish to abandon this way of life considering the false sense of freedom^15^. The difficulty in maintaining affective ties with the family and non-family members combined with the factors mentioned above contribute to discontinuation of treatment of TB^16^.

Health care is usually of minor concern for homeless people because survival is a primary necessity as a result of the lack of resources for treatment and continuous exposure to violence^17^. In addition, the biomedical model prevailing in healthcare services cannot adequately respond to the healthcare needs of this social group^18^.

The meanings attributed to the control of TB indicated the importance of street clinic teams helping this population by improving access to health services and social equipment and referring patients to other social and health services. A survey carried out with street clinic teams involved in caring for people with TB indicated that they significantly helped increase access to health services, early diagnosis and treatment, and follow-up until a cure was achieved^5^.

Teamwork is fundamental in health care because of the singularities and complexities of street dwellers, evidencing that the articulation between different areas of knowledge, care practices, and subjects results in the development of interventions appropriate to the needs of this group ^(^ ^19^. The care model used in street clinics increased the access to health care and allowed adequate and personalized care to socially marginalized people, who recognized this modality as a reference health service^20^.

In addition, the characteristics and behavior of street people make them susceptible to social stigma and rejection, reflected by discrimination by those who should provide care, and this condition limits access to health services and affect the diagnosis and treatment of TB, further aggravating the condition of this population.

Respondents indicated that they faced resistance from street dwellers in the first contacts; however, over time, bonds were established, allowing providing health care and other services required by this population. In addition, art workshops favored dialogic relationship and qualified listening, which helped establish bonds and implement actions aimed at mitigating damage^21^.

One of the factors that helped provide health services was the flexibility of health professionals to provide care when street people sought health services. However, the context of vulnerability and precariousness, prejudice, discrimination, and the need for professional care during unpredictable situations prevent developing these interventions.

The limited access to health services confirm the findings of an integrative review^22^ that associated these difficulties with social stigma and prejudice both because of the behavior of this population, including verbal or physical aggressiveness, and appearance, including clothing and lack of hygiene.

The testimonies indicated that the knowledge of street dwellers about the signs and symptoms of TB favored early diagnosis; however, the restricted access to health services limited diagnosis. The difficulty of this group in adhering to TB treatment was primarily due to personal factors such as physical characteristics and lifestyle.

In the perception of the professionals from street clinic teams, the denial of the disease stems from the fear of transmitting the disease or being excluded from the group, knowledge of the possibility of treatment failure, and the need to decrease the use of illegal drugs during treatment.

The lack of a life project was one of the determinants of nonadherence to TB treatment. The expression of negative feelings by street people reveals that life is no longer meaningful and valuable, mainly because of the difficulties in living on the street and the lack of prospect of a promising future^23^. A study involving the street population of the central region of São Paulo on the meanings and perceptions related to TB and DOT found that the risk of discontinuing treatment was high because of social factors, including physical and moral violence, stigma over the disease, use of legal and illicit drugs, and knowledge of the disease^2^.

People on the street are more vulnerable to treatment discontinuation because adherence is complex and depends on factors related to people with TB and health services. The factors that may compromise individual treatment include side effects, prolonged treatment time, improved clinical status after the onset of tuberculostatic use, poor living conditions, absence of fixed housing, drug abuse, lack of knowledge of the disease, non-acceptance of diagnosis, and the presence of other associated diseases. Other reasons include the lack of preparation of health professionals for providing care to this population and the difficulty in establishing a bond between health professionals and people with TB. Prejudice against this social group hinders access to health services for supervised treatment or medical consultation^24^.

The professionals consider that the provision of meal vouchers, partnership with reception centers, hospitalization in rehabilitation hospitals during treatment, and enrollment of street dwellers in professional courses may help provide care to individuals with TB and improve adherence to treatment.

The prevalence of TB in homeless people is high. Therefore, health professionals should evaluate different treatment strategies, from the active search of individuals with respiratory symptoms to the provision of care for people with TB.

The control of TB in this population involves several conditioning factors because the access to medicines and health services is not enough to guarantee adherence to treatment. The factors considered essential were the understanding of the health-disease process as a social phenomenon, acknowledgment of the health needs in coping with TB with a focus on patient acceptance, and the responsibility of health professionals in caring for this social group.

The health condition of this population is a cause for concern and a significant challenge in view of the scope, increase in the rate of morbidities, and high number of adversities, which requires the promotion of intersectoral actions because this problem involves political, economic, cultural, and social factors^25^. In this respect, the Theory of Social Determination of the Health-Disease Process has significantly contributed to understand and treat TB in street dwellers because it is necessary to abandon the health care models that disregard this process as a social phenomenon and do not correlate their occurrence with social factors.

The limitations of the present study were the collection of data in only one PHCU, which limited the sample size. In addition, the activities that targeted this population are only part of the work carried out by the street clinics of São Paulo.

## Conclusion

According to the opinions of the interviewed professionals, health care for homeless people is a socially relevant issue because their living conditions, marked by many adversities, including risk factors and limitations in access to health services and treatment, making them more vulnerable to illness, discontinuation of treatment, and death from TB. Therefore, the actions of health professionals trained to meet the needs of this group may help control the disease. However, TB in this group is a health problem and a challenge for health managers and professionals and consequently needs to be addressed appropriately to subsidize the formulation, implementation, and use of health technologies consistent with the reality of street people.

Therefore, the findings of this study should raise awareness about this mode of care with a focus on TB. It is important to highlight the contribution of the study to the professional practice of nurses, expanding their field of practice, and considering that these professionals improve the health care to people living in the streets. For this purpose, it is necessary to increase awareness about this topic and invest in training professionals capable of dealing with a complex reality involving biopsychosocial and spiritual factors associated with the condition of living in the streets.

## Footnotes

1 Printed material: São Paulo. Health Surveillance Coordination. Center for Disease Control. Tuberculosis Control Program. Tuberculosis Bulletin of the City of São Paulo 2016. São Paulo: COVISA; 2016.

## Footnotes

2 Study developed in the municipality of São Paulo and published as a book chapter: Souza ES, Zioni F. Directly observed treatment for tuberculosis among the street population. (O tratamento diretamente observado para a tuberculose entre a população em situação de rua.) In: Rosa AS, Brêtas ACP. Nursing and health: Insights into the street situation. (Enfermagem e saúde: olhares sobre a situação de rua.) Curitiba: CRV; 2013.
